# Social Ultrasonic Vocalization in Awake Head-Restrained Mouse

**DOI:** 10.3389/fnbeh.2016.00236

**Published:** 2016-12-19

**Authors:** Benjamin Weiner, Stav Hertz, Nisim Perets, Michael London

**Affiliations:** Edmond and Lily Safra Center for Brain Sciences and Life Science Institute, The Hebrew University of JerusalemJerusalem, Israel

**Keywords:** courtship ultrasonic vocalizations, USV, social interaction, vocal communication, song syntax, head fixation, stress

## Abstract

Numerous animal species emit vocalizations in response to various social stimuli. The neural basis of vocal communication has been investigated in monkeys, songbirds, rats, bats, and invertebrates resulting in deep insights into motor control, neural coding, and learning. Mice, which recently became very popular as a model system for mammalian neuroscience, also utilize ultrasonic vocalizations (USVs) during mating behavior. However, our knowledge is lacking of both the behavior and its underlying neural mechanism. We developed a novel method for head-restrained male mice (HRMM) to interact with non-restrained female mice (NRFM) and show that mice can emit USVs in this context. We first recorded USVs in a free arena with non-restrained male mice (NRMM) and NRFM. Of the NRMM, which vocalized in the free arena, the majority could be habituated to also vocalize while head-restrained but only when a female mouse was present in proximity. The USVs emitted by HRMM are similar to the USVs of NRMM in the presence of a female mouse in their spectral structure, inter-syllable interval distribution, and USV sequence length, and therefore are interpreted as social USVs. By analyzing the vocalizations of NRMM, we established criteria to predict which individuals are likely to vocalize while head fixed based on the USV rate and average syllable duration. To characterize the USVs emitted by HRMM, we analyzed the syllable composition of HRMM and NRMM and found that USVs emitted by HRMM have a higher proportion of USVs with complex spectral representation, supporting previous studies showing that mice social USVs are context dependent. Our results suggest a way to study the neural mechanisms of production and control of social vocalization in mice using advanced methods requiring head fixation.

## Introduction

Vocalizations are a part of natural mouse behavior (Eric Hill, 1944; Sewell, 1968). When a male mouse encounters a female, the male enacts a courtship behavior which includes emission of vocalizations in the ultrasonic frequency range. The high temporal correlation between the ultrasonic vocalizations produced by male and female mice indicates that they play a role in social interactions and courtship behavior (Neunuebel et al., 2015). These ultrasonic vocalization (USV) syllables mostly consist of single or dual narrow-band frequency chirps with rapid frequency jumps, creating complex spectral structure (Holy and Guo, 2005; Egnor and Seagraves, 2016; Matsumoto and Okanoya, 2016). Several studies have found that mice modify their syllable content in response to different cues and social situations (Yang et al., 2013; Chabout et al., 2015; Mun et al., 2015; Gaub et al., 2016; Grimsley et al., 2016; Seagraves et al., 2016).

The combination of a sophisticated set of genetic tools available in the mouse alongside the ultrasonic vocalization behavior makes the mouse an attractive system for understanding mammalian vocal control and dysfunctions (e.g., stuttering) as well as social interaction and social disorders. Several studies have used direct and genetically driven lesions, vocalization driven activity-dependent genes, neural tracing and histology, and cross-species anatomical comparison to identify key brain regions responsible for USV production and control (Arriaga et al., 2012; Pfenning et al., 2014; Hammerschmidt et al., 2015). Yet, direct electrophysiological and optical recordings from relevant brain areas during production of USVs have not been performed.

Some of the most useful advanced neuroscience methods such as optical recordings of identified neuronal population activity using two-photon microscopy or intracellular whole-cell recordings are possible in the awake and behaving mouse (Crochet and Petersen, 2006; Nguyen et al., 2009; Komiyama et al., 2010). However, these methods currently require the mouse to be head-restrained to achieve mechanical stability, which could produce elevated stress levels and may disrupt social behavior. It is well-known that stress can be a limiting factor when observing natural behavior. For example, marmosets are only able to emit a single call type while head-fixed (Eliades and Wang, 2005). In rats, vocalizations in a stressful situation predicted both performance in spatial learning and resilience to future stress (Drugan et al., 2014). Likewise, mice also emit fewer spontaneous and social interaction related vocalizations while restrained or after stressful manipulation (Lumley et al., 1999; Chen et al., 2013; Chabout et al., 2015). Perhaps for these reasons, electrophysiological and optical recording (and stimulation) approaches have not yet been applied to explore USV production and control in mice during social encounters.

In order to enable future studies of the neural basis of USV production and control using electrophysiology and two photon imaging, we carried out a systematic study to determine the conditions under which male mice produce social USVs while head-restrained. To this end, we report here a head-fixation method to allow mice to walk or run and interact with female conspecifics and quantify the similarities and differences of social USVs emitted during head fixation in comparison to those emitted by non-restrained mice.

## Materials and methods

### Animals

We used 17 C57BL/6 male and 10 female mice (8–12 weeks old). All mice were group housed (3–4 per cage) and kept on a 12 h light (7 a.m.–7 p.m.)/dark cycle with *ad-libitum* food and water.

### Ethical note

This study and the procedures it includes are stage one of a more comprehensive project which was approved in ethical permission NS-16-14216-3 entitled “Functional identification of brain regions involved in ultrasonic vocalization in mice,” approved by the Hebrew University Animal Care and Use Committee under the Israel Act for animal experimentation 1994. All surgical procedures are performed under anesthesia and postoperative care is administrated to reduce pain. Mice recover quickly from the procedure and we could not identify by observation any difference in their behavior. Recording session 2 was designed to evaluate whether there are any differences in the vocalization behavior after recovering from surgery. We have not identified any such signs either in vocalization rate or other parameters. The severity of the request was set to level 3 according to the Israel Act for animal experimentation 1994. Severity level 3 requires animal monitoring twice a day after surgery for 3 consecutive days and postoperative care. After 3 days, the animals were monitored twice a week for the duration of the experiments.

### Recording apparatus

For ultrasonic vocalization recording we used an UltraSoundGate system (Avisoft bioacustic, Germany) composed of a CM16/CMPA ultrasound microphone, UltraSoundGate 116H computer interface, and USGH recorder software on a standard PC computer. Sampling frequency of 250 KHz and 16-bit recordings were used. For online monitoring we used simultaneous display of the spectrogram (256 points FFT). The microphone was placed above the center of the cage in the free arena and 5–20 cm from the head-fixed mouse and a USB web camera was used to record video of the behavior before and during the encounter.

### Recording sessions in free arena

**Figure 1:**
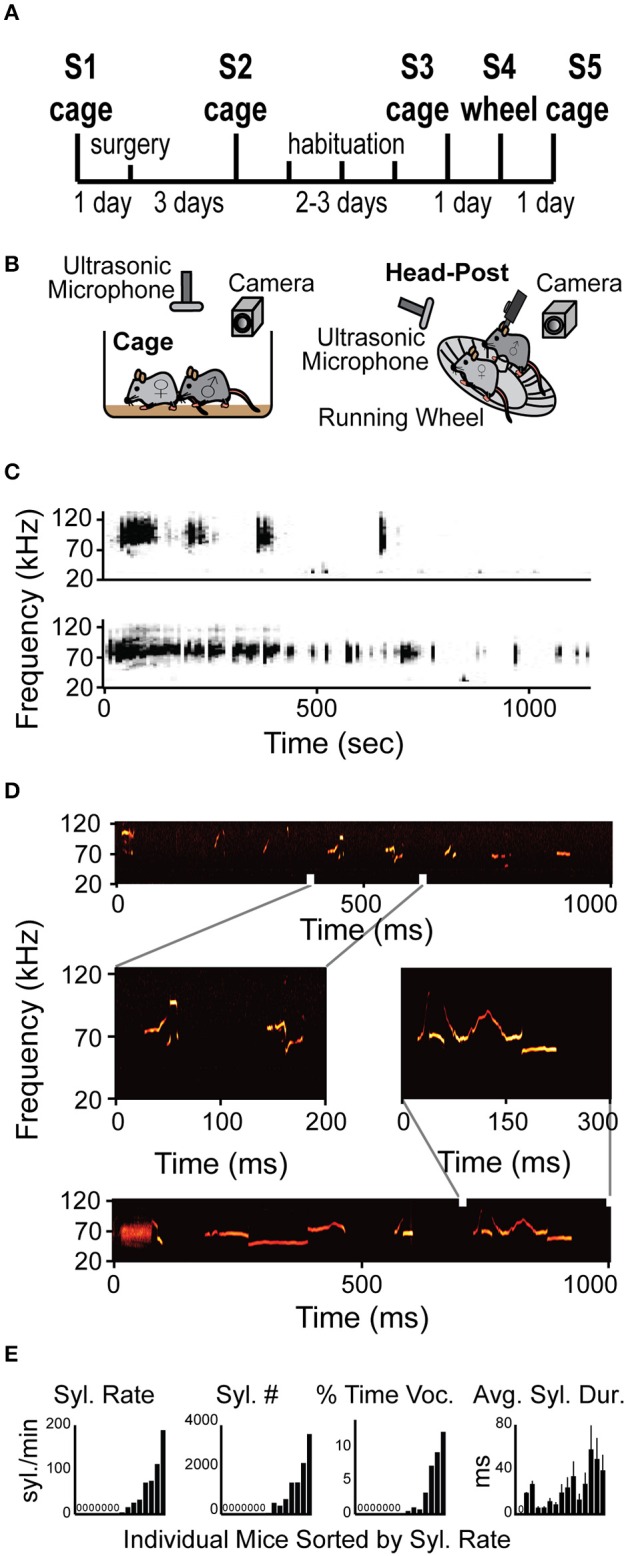
**Mice vocalize when head-fixed. (A)** Diagram of the experimental design. The protocol included recordings in arena (cage) and one session recording head-fixed (wheel) (See Section Materials and Methods for details). **(B)** Schematic illustration of the experimental apparatus while non-restrained (cage) and head-restrained (head-post). Male is head-fixed while female is freely moving on the running wheel. **(C)** Representative spectrogram examples of two entire vocalization sessions from two different HRMM × NRFM interactions. **(D)** Examples of several different types of syllables within a single session of HRMM vocalizations. Zoomed in vocalizations examples demonstrate complex vocalizations. **(E)** Quantification of vocalizations rate, total number, time, and duration for individual mice. All graphs are sorted by lowest to highest syllable rate (*n* = 17 mice).

After 5 min of handling (standard for behavioral experiments), non-restrained mice were placed in a new housing cage, which had been autoclaved and contained fresh bedding. This was done to avoid any scent carryover between different mice. Each mouse was placed in a fresh cage for 5–10 min for habituation. Subsequently, we started recording sounds while a novel female was introduced into the cage and remained for 15 min. Every encounter was with a novel female, however different males did encounter the same female but never in close temporal proximity. During this time the male carried out the standard mating ritual which has been documented thoroughly (McGill, 1962). The female was then removed if the male showed interest and did not vocalize, however if the male continued to vocalize the female was left in the cage for an additional 4 min. Recordings were continued for one additional minute after the female was removed from the cage. All other animals were removed from the room during recording. All recordings were performed in the morning (light phase from 7 a.m.–2 p.m.) to reduce the effects of the females' estrus cycle. We performed recordings at each stage of the procedure to determine whether any manipulation had an effect on vocalizations (Figure 1A). A non-restrained male mouse (NRMM) was introduced to a non-restrained female mouse (NRFM) in the first social interaction session (session 1) and their baseline vocalization behavior was recorded. Following that session they underwent head-post implantation surgery and recovery. Another social interaction session followed to assess changes in behavior due to surgery (session 2). Mice then went through three rounds of habituation to the head fixation apparatus. During this time no NRFM was introduced. The next session (3) assessed changes in USV production due to the habituation process (this session was identical to session 1 with free arena NRMM and NRFM). On session 4 (test session) mice were attached to the head-fixation apparatus and after 5 min an NRFM was introduced (Figure 1B). The estrus stage was determined (McLean et al., 2012), revealing that 31% of recordings occurred with a female in estrus or proestrus. However, a previous study found no correlation between the number of syllables emitted and female estrus state (Kim et al., 2016).

### Surgery

On the same day, or 1 day after the first vocalization recording session, mice were implanted with a head-post for head restraint. Surgeries were performed under isoflurane anesthesia. Rymadil analgesia (10 mg/kg body weight, 200 μl injection volume) was administered prior to incision of the skin. After skull cleaning, a small 3 × 3 × 15 mm metal head post was attached to the skull using Cyanoacrylate glue and MetaBond dental cement (Sun Medical). Immediately post-surgery, animals were placed in a ventilated cabinet with warm air adjusted to 34°C, containing drinking water only. Animals were monitored every 30 min until they appeared to recover by visual inspection. Animals were considered recovered from anesthesia when they maintained themselves upright and able to move purposefully. Mice were administered another doze of analgesia (Rymadil, 10 mg/kg body weight, 200 μl injection volume) 12 h post-surgery and monitored for 3 consecutive days post-surgery. No weight loss or other clinical signs were detected.

### Head restrained recording sessions

After recovery, another recording session was made to measure any effects of surgery on vocalizations. The mice were then habituated to the head-fixing apparatus, which consisted of a plastic running wheel (BioServ, USA) and a custom-made clamp mounted on an articulating arm (Noga holding systems, Israel). We found that adjusting the head position relative to the wheel individually for each mouse helps with the habituation process. In total, the habituation time was at least 3 h (cumulative) and no more than 6 h, spread as 30 min sessions over a few days. The mice were then placed in a cage with a novel female to determine the effects of habituation on vocalizations. 24 h after this recording session, these head-fixed mice were placed on the running-wheel. Five minutes later, a female was placed on the wheel alongside the head-fixed mouse and allowed to move freely. Mice were not able to mount the female but were able to bring the female closer by running on the wheel, as the female was un-restrained. During the next 20 min, vocalizations were recorded, and then both mice were removed from the apparatus. 24 h later, vocalizations of the non-restrained male were recorded again with a new female in the cage to detect any lingering stress effects of head fixation. Mice were considered habituated when they were grooming, running on the wheel and did not show freezing behavior.

## Data analysis

**Figure 2:**
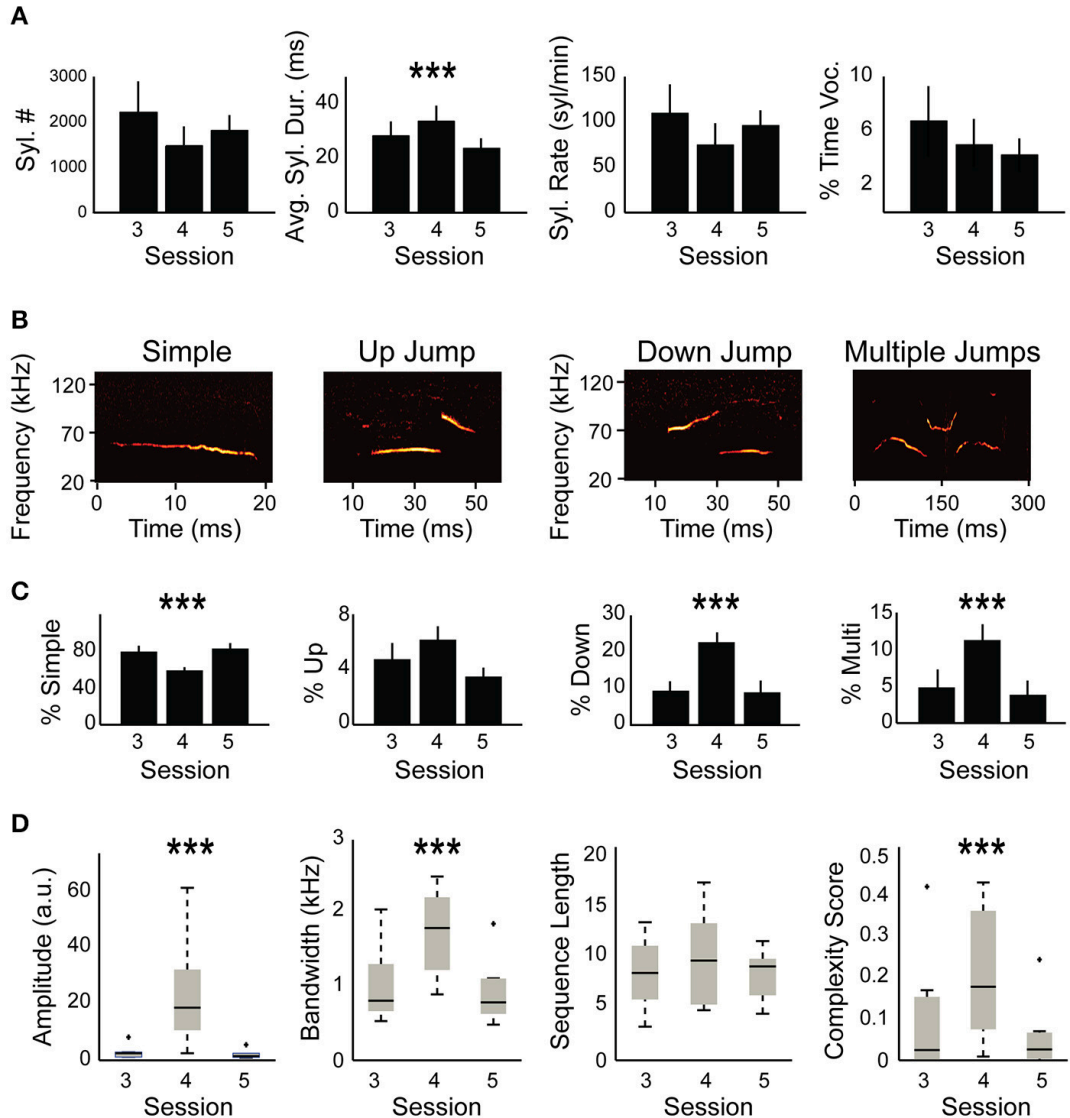
**Syllable composition is different between non-restrained and head-restrained sessions. (A)** Quantification of USVs including: total number of syllables, syllable duration, syllable rate, and fraction of time during USVs of the total session (in %). The averages of these parameters are compared between the head-fixed session (S4) and the session before (S3) and the session after (S5). Only average syllable duration was longer due to head restraint (*p* = 0.0005). **(B)** Spectrogram example from each syllable category including “simple,” “down,” “up,” and “multiple.” **(C)** Distribution of syllable types between the different sessions. A decrease in “simple” syllables in the head-restrained session (S4, *p* < 0.0001) is accompanied by an increase in “down jump” and “multiple jump” syllables (*p* < 0.0001) and “multiple jumps” syllables (*p* = 0.0003) while no change occurred in “up jump” syllables (*p* = 0.058). **(D)** Amplitude (*p* < 0.0001), bandwidth (*p* < 0.0001) and complexity (*p* = 0.0005) were affected by head restraint whereas sequence length (*p* = 0.12) did not show any significant differences. ^***^p < 0.001.

The analysis of the data was done using “Mouse Analyzer v1.3” program written in MATLAB by Holy and Guo (2005) and modified by Chabout et al. (2015), and available online (http://jarvislab.net/research/mouse-vocal-communication/). The software extracts a list of syllables from each recording. We used a default white noise threshold of 0.3 and 256 samples/block with half overlap which matched a manual counting of syllables for several files. The frequencies outside of USV song range under 20 KHz were truncated. Our criteria were a default of 10 ms minimum separation between syllables and 3 ms for the shortest detectable syllable. The syllables were classified into four different categories according to their pitch jumps (Figure 2B). Syllables with no pitch jump were classified as “simple” syllables. Syllables with one pitch jump were classified to the “up” or “down,” depending on the direction of the jump. Syllables with more than one pitch jump were classified as “multiple.” In addition, syllables that did not fit into these categories were “unclassified” and constituted 0.5 % of all syllables. Each extracted syllable is thus characterized by its type, duration, ISI (inter-syllable interval) as well as frequency and amplitude properties. Some of our recordings contained a stable noise in a single frequency band. The intensity in that frequency was reduced prior to the analysis using “sox” auditory processing toolbox (http://sox.sourceforge.net/) in order to obtain a more accurate analysis. In total across all sessions, we analyzed 81,561 syllables from 17 males (2 males only underwent the first session due to lack of vocalizations) interacting with 10 females. We used a single microphone and therefore cannot separate male vs. female vocalizations. For the first session *post-hoc* prediction, we analyzed only the first 10 min to reduce variability in recording times.

### Statistical analyses

Summary data are expressed as mean ± SEM unless otherwise stated. They are reported in the Results Section and figure captions together with their corresponding significance level. The data collected in sessions 3, 4, and 5 were used to test the effect of head fixation on various vocalization parameters. We used R (R core team, 2012) and its linear mixed effect package lme4 (Bates et al., 2015) to perform a linear mixed-effect models on the relationship between syllable rate (outcome variable) and head fixation (and similarly for total syllables emitted, syllable duration, and % time spent vocalizing; Figure 2A). As fixed effects, we entered head fixation into the model. As random effects, we added mouse identity. Visual inspection of residual plots did not reveal any obvious deviations from normality. *P*-values were obtained by likelihood ratio tests of the full model with the fixed effect in question against the model without the fixed effect in question. We ran a similar analysis for the percentage of the four various types of syllables (Figure 2C). The same analysis was also applied to the vocalizations average amplitude, average bandwidth, average sequence length, and average sequence complexity score (Figure 2D). To obtain parameters for predicting head-restrained male mice (HRMM) vocalizers, Student's non-paired *t*-test was used to compare HRMM vocalizers and non-vocalizers on recordings from session 1. Corrections for multiple comparisons were applied as in Benjamini and Hochberg (1995). For graphical purposes, statistical significance was denoted as ∗, ∗∗, or ∗∗∗ for original *p*-value thresholds of 0.05, 0.01, and 0.001 respectively (and the FDR corresponding thresholds were used in the test). Correlations were computed using standard Pearson linear correlation test.

## Results

### Head-restrained male mice produce ultrasonic vocalizations

In order to test if HRMM would show USV courtship behavior when head-fixed and to characterize this behavior, we designed a protocol for gradual habituation (Figures 1A,B and Methods). Briefly, during the protocol mice were introduced to a female in three sessions (S1–S3, Figure 1A) and their vocalizations were recorded. Mice were then adapted gradually to the head-fixation apparatus. On the fourth session (S4, Figures 1A,B) HRMM vocalizers immediately responded to a NRFM by running on the wheel and emitting sequences of vocalizations indistinguishable, upon online visual inspection of the spectrogram, from their normal non-restrained behavior (Figures 1C,D). HRMM emitted no USVs in session 4 before the female was introduced. Quantitative analysis has shown that the basic properties of individual syllable types including the inter-syllable interval distribution and the average USV sequence length had no significant difference from their corresponding values in the first session (NRMM) (Supplementary Table 1). This finding, combined with the observation that HRMM were never vocalizing unless a female mouse was present, suggest that the HRMM show social USV behavior. Seven out of 15 mice were head-fixed vocalizers emitting 1477 ± 435 (mean ± SD) syllables during the encounter session of 15–20 min (average 18.33 min) resulting in 75 ± 23 syllables per minute (Figure 1E). The other eight head-fixed mute mice were almost completely silent (producing 9 ± 6 syllables for the whole session). We quantified the percentage of time mice spent vocalizing and found that head-fixed vocalizers emitted 51.3 ± 17.5 ms of vocalizations per second (about 5% of the entire session). Notably, in the first 5 min of the session the percent time vocalizing went as high as 27%. The average individual syllable duration across vocalizing mice was 35.4 ± 5.9 ms. Lastly, the median inter-syllable interval for all sessions was 92.2 ms (with no difference between sessions) which is slightly longer than previously reported in mice freely moving in an arena (Grimsley et al., 2011).

### Syllable composition is different between free arena (NRMM) and head-fixed (HRMM) sessions

Mice were previously shown to change their vocalization pattern in different contexts (Chabout et al., 2012, 2015; Yang et al., 2013; Mun et al., 2015; Srivastava et al., 2015; Gaub et al., 2016; Grimsley et al., 2016; Heckman et al., 2016; Hoier et al., 2016; Seagraves et al., 2016). We therefore hypothesized that head-fixation likely modulates USVs in comparison to non-restrained sessions. To test this hypothesis, we compared the basic parameters described in Figure 1E between restrained and non-restrained sessions. Statistical analysis using mixed effect models (see Section Materials and methods) did not reveal a significant effect of head fixation on total syllables emitted, syllable rate, or the percent of time the mice spent vocalizing [$\chi_{\left(1\right)}^{2}$ = 1.61, *p* = 0.2, $\chi_{\left(1\right)}^{2}$ = 1.66, *p* = 0.19, $\chi_{\left(1\right)}^{2}$ = 0.047, *p* = 0.82, respectively; Figure 2A]. However, head fixation affected syllable duration [$\chi_{\left(1\right)}^{2}$ = 11.9, *p* = 0.0005], increasing it by 9.2 ms ± 2.1 (standards errors). We suspected that this change in syllable duration is likely related to a potential change in the distribution of different syllable types due to head fixation. We therefore performed a similar test for each of the four different syllable types. The analysis showed that head fixation reduced the percent of simple syllable by 23.1% ±2 [$\chi_{\left(1\right)}^{2}$ = 11.9, *p* < 0.0001]. The proportion of “down” syllable increased by 13% ± 1.75 [$\chi_{\left(1\right)}^{2}$ = 15.4, *p* < 0.0001] and so did the percentage of “multiple” syllables +7.2% ± 1.1 [$\chi_{\left(1\right)}^{2}$ = 13.2, *p* = 0.0003]. On the other hand we did not find a significant effect for the proportion of “up” syllables [$\chi_{\left(1\right)}^{2}$ = 3.6, *p* = 0.058], possibly due to the low sample size of “up” syllables (Figure 2C). We have also noticed an increase in sound amplitude during the head-fixed session which was probably due to the closer distance of the mouse to the microphone [$\chi_{\left(1\right)}^{2}$ = 23.2, *p* < 0.0001, Figure 2D]. Bandwidth of syllables also increased in the HRMM condition by 9.1 KHz ±1 [$\chi_{\left(1\right)}^{2}$ = 28.4, *p* < 0.0001], signifying a greater frequency range in syllable repertoire. Lastly, while we have not found a significant change in the average sequence length [$\chi_{\left(1\right)}^{2}$ = 2.35, *p* = 0.12], we have found a significant increase in the complexity score [0.15 ± 0.04; $\chi_{\left(1\right)}^{2}$ = 12.0, *p* = 0.0005, complexity score analyzed as in (Chabout et al., 2015)]. This increase is in line with the increase in the proportion of “multiple” syllables in the head restrained condition (Figure 2C right panel).

### Number of vocalizations in free arena (NR) sessions does not predict HRMM vocalizers

**Figure 3:**
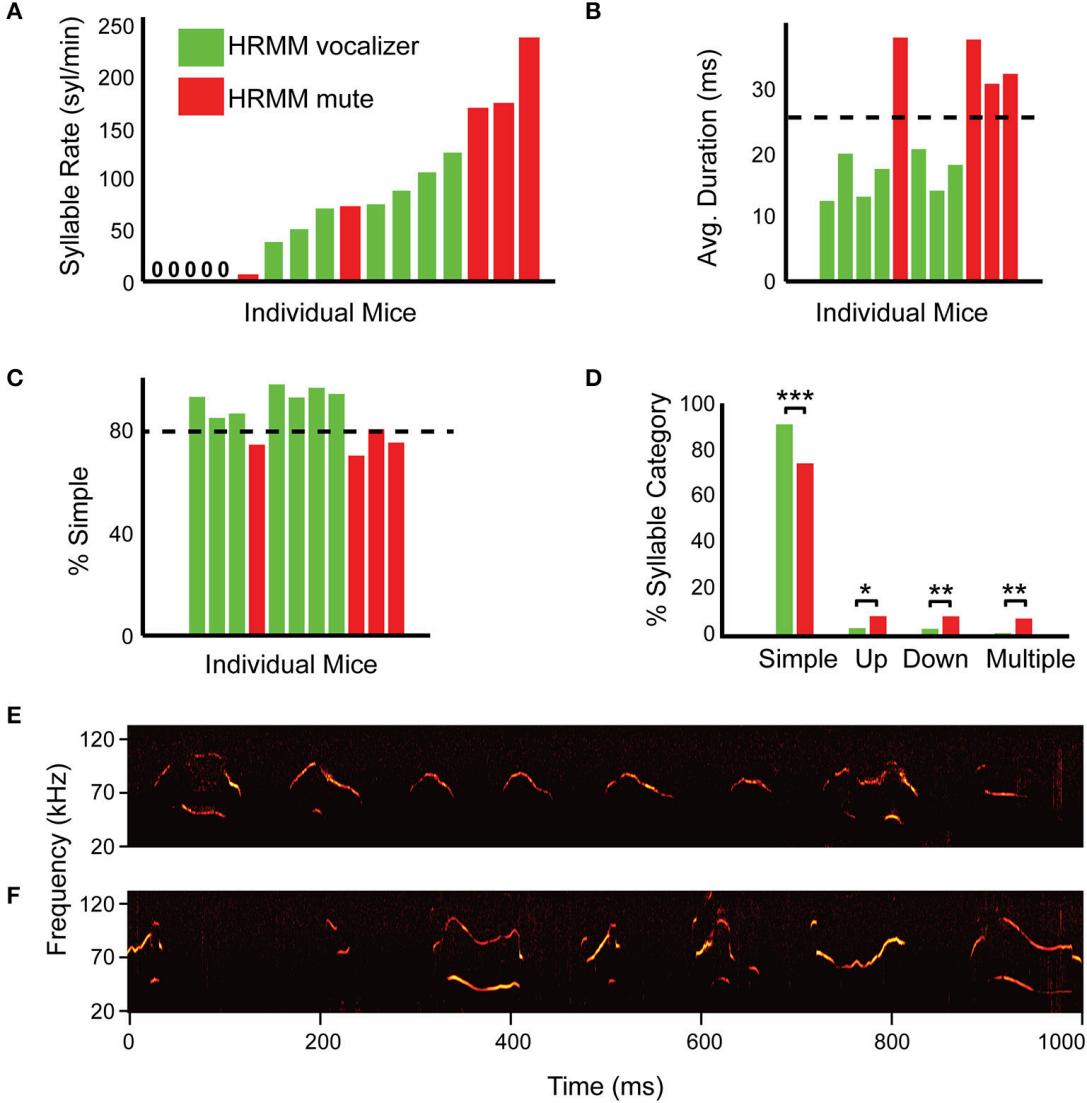
**Simple percentage and average syllable duration successfully predict head-fixed vocalizers. (A)** Quantification of syllable rate for individual mice in the first session (S1—non-restrained male and female) colored by their performance on session 4 (head restrained). Green marks mice that vocalized when head restrained and red marks mice that were mute when head-fixed. Note that some mice vocalized at high rate in session 1 but nevertheless after habituation were mute when head-restrained. This indicates that syllable rate alone is insufficient as a predictor for head-restrained vocalizations. **(B)** Average USV durations sorted by syllable rate for each individual head-restrained vocalizing mouse in session 1 with 11/17 total mice represented (color code as in **(A)** with head-restrained mute mice omitted). Head-restrained vocalizers made shorter duration syllables on average as compared to head-restrained mute mice (*p* = 0.0001). **(C)** Percentage of “simple” syllables for each individual head-restrained vocalizing mouse during session 1 sorted by syllable rate (same color code as in **B**). **(D)** Comparisons of syllable percentage across syllable category for HRMM—vocalizers and mute. “Simple” syllables (*p* = 0.0002) can assist in predicting which mouse can be habituated to vocalize while head-fixed. **(E)** A representative example of a spectrogram from HRMM vocalizer in first session (S1, while non-restrained) with few “non-simple” vocalizations. **(F)** A representative example of a spectrogram from first session mute HRMM with many “non-simple” vocalizations. ^*^p < 0.05, ^**^p < 0.01, and ^***^p < 0.001.

Not all male mice vocalized equally. We were interested in developing a way to predict, in advance, if a mouse would vocalize when head-fixed. We examined vocalizations from session 1 to develop a prediction method to assist in the selection of mice with a high potential for head-fixed vocalizations before going through the head-post implantation and the habituation schedule. All head-fixed vocalizers showed a high (>50 USVs/min) vocalization rate during the first NRMM session except a single mouse vocalizing at 40 USVs/min (Figure 3A). However, three head-fixed mute mice had a high vocalization rate on session 1. In addition, none of the mice that vocalized less than 20 syllables per minute in session 1 vocalized head-fixed. We therefore conclude that a vocalization rate greater than 20 USVs/min in the first session is the first criterion for selecting HRMM vocalizers, but is not a sufficient predictor.

### Average syllable duration and “simple” syllable percentage predicts head-fixed vocalizers

Another property of the vocalizations that we tested as a predictive feature was the average syllable duration and the relative prevalence of different syllables types (Figure 2B). We examined the ratio of these categories in the first session recordings of each of the mice (Figures 3C–F). Head-fixed vocalizers in the first session had significantly shorter average syllable duration (HRMM vocalizers, 16.4 ms, HRMM mute: 34.4 ms, *T* = 8.47, *p* = 0.0001), more simple syllables (HRMM vocalizers, 91.9%, HRMM mute: 75%, *T* = −6.0, *p* = 0.0002) and fewer up-jump (HRMM vocalizers, 3.6%, HRMM mute: 8.7%, *T* = 2.53, *p* = 0.0321), down-jump (HRMM vocalizers, 3.2%, HRMM mute: 8.7%, *T* = 3.64, *p* = 0.0054), and multi-jump syllables (HRMM vocalizers, 1.4%, HRMM mute: 7.7%, *T* = 4.0, *p* = 0.0029) (Figures 3B–D). We determined that the second inclusion criterion is more than 80% of simple syllables in the first session and average syllable duration below 25 ms (Figures 3B,C). We predict that using these combined criteria will reduce the percentage of mute HRMM.

## Discussion

### Mice can emit social vocalizations when head-restrained

We have demonstrated for the first time that mice are able to emit social vocalizations when they are held head-restrained on a running wheel. After habituation, 7 mice out of the 11 mice that were vocalizing in the free arena were also vocalizing when head-restrained. This sets a success rate of at least 60% if all mice undergo the habituation protocol. A few studies have indicated that mice and rats can produce head-restrained sonic and ultrasonic spontaneous vocalizations, however this is the first systematic demonstration of head-restrained social interaction related vocalizations in mammals (Reed et al., 2013; Grimsley et al., 2016). Because not all mice vocalized, we tested several strategies to increase the probability of head-fixed vocalizations but none of these manipulations seemed to be effective. Several manipulations have been used in the past to increase vocalization performance including extended habituation and overnight female experience (Grimsley et al., 2011; Hanson and Hurley, 2012; Chabout et al., 2015; Ferhat et al., 2015; Heckman et al., 2016). In our hands, however, using these and additional methods on non-vocalizers produced little change. However, due to the small number of non-vocalizing mice included, it is impossible to draw strong conclusions. We therefore carefully suggest that the factors affecting head-fixed social interaction related vocalizations such as stress susceptibility and social status may be quite rigid, but further investigation into this is required (Low et al., 2016). Our longitudinal recordings along the timeline of our experiment suggest that the basic social vocalization behavior is very robust. We have monitored the vocalizations at various time points (after head-post implantation, after habituation, and after encounter with female while male is head-fixed) and concluded that these procedures had no significant lasting effect on the vocalization behavior as measured in terms of USV rate, number or composition. However, the syllable composition did change in the head-restrained session, while basic properties of the vocalization such as syllable rate, inter-syllable interval distribution and sequence length were not significantly different (Supplementary Table 1). We interpret these results as an indication that the vocalization behavior is sensitive to context as previously shown (Chabout et al., 2012). Importantly, HRMM were unable to mount and perform the normal mating ritual (see Supplemental Movies) which could contribute to the changes in some parameters of the vocalizations.

### Predicting which mouse is a head-fixed vocalizer

In order to facilitate a prediction of which mouse has a high probability to vocalize when head-fixed, we analyzed our data and chose our prediction criteria to include the successful vocalizers post-hoc. Clearly this procedure does not guarantee a 100% success rate, and we cannot rule out the possibility that some mice may vocalize head-fixed even without meeting these criteria and vice versa. Nevertheless, it is interesting that vocalizations emitted by a mouse on its first encounter with a female have some predictive power regarding its future behavior. It suggests that interesting information about the individual mouse behavior is contained in its vocalizations. One confounding factor for further investigation of this finding is an appropriate method for syllable classification and sequence analysis. The analysis tools we have used, based on (Chabout et al., 2015), while useful and efficient, are oversimplified to capture the uniqueness and variability between even a single animal's vocalizations during a single session. For example the “Simple” class, which includes USVs with no pitch jump, can include very short syllables (~5 ms duration) as well as very long ones (>100 ms), which are clearly distinct in the spectrogram display. Several categorization schemes have been proposed (Holy and Guo, 2005; Panksepp et al., 2007; Scattoni et al., 2008; Fischer and Hammerschmidt, 2011; Sugimoto et al., 2011; Hanson and Hurley, 2012) and have varying degrees of success in capturing the richness of the vocalizations expressed during courtship. However, there is an urgent need for objective and automated analysis tools with higher resolution and sensitivity to syllables structure. The recent success in computer understanding of human speech (Amodei et al., 2016) indicates that such tools are within reach. Yet, in contrast to human speech, where sound can properly be labeled into meaningful syllables, the lack of such ground-truth labeling in the case of USVs makes automated classification particularly challenging.

### Social interaction related vocalizations as a benchmark of stress

What makes some mice vocalize and some not? One option is the tonic level of stress. Stress serves essential functions in biological systems. Our application of vocalization analysis to the prediction of which mouse will vocalize can be interpreted also in that context (Manteuffel et al., 2004; Briefer, 2012). It suggests that the USV system, even under natural interaction situations (when both male and female are non-restrained), might indicate that mice have different tonic stress levels and that their vocalizations may be a useful way to quantify them. While several consistent and classical tests of stress exist, the vocalization system is both a complex and interesting behavior, which can also be used as a proxy for stress without directly stressing the animal (only by introducing a female). It is important to note that these social interaction related vocalizations are in the ultrasonic range, while stress related vocalizations are in the sonic range (Grimsley et al., 2016). However, we show that the social interaction related vocalizations are also sensitive to situations in which stress is a key factor. Therefore, further studies can look at the effect of various stress factors on vocalization while measuring conventional physiological parameters of stress (e.g., heart rate, respiration rate, neuroendocrine activity) to clarify their presence and determine whether they modulate vocalizations in a meaningful way. This will show whether nuances in vocalization are sensitive enough to indicate the level of stress. Recently many experimental setups have adopted head-fixation in mice to study simple and complex behaviors. However, tonic stress level, which could be a confounding factor in many of these behaviors, is often not quantified as mice produced no stress related sonic vocalizations while head restrained. It is currently unknown how different platforms for head-fixation such as a tube, a floating ball, a running wheel, a treadmill or a floating cage (Crochet and Petersen, 2006; Dombeck et al., 2007; Niell and Stryker, 2010; Kislin et al., 2014; Vinck et al., 2015) affect the stress level of the subject mouse. It would be interesting to test which of these methods would permit social interaction related vocalization emission following habituation. Moreover, the observation that head restrained mice interact socially opens the door for further research to elucidate the neural mechanisms of social behavior under highly controlled conditions.

### Suitability of head restrained mice to study social interactions

The use of head restraint has become a popular tool for studying brain activity in sensory and motor systems of awake rodents using advanced methods such as two photon microscopy and intracellular recordings. Clearly, the advantage of well-controlled conditions comes at the price of unnatural behavior of head restrained animals. Therefore, it is expected that applying head restraint protocols to study brain activity during social behavior would be challenging because the animal needs to be at a state which enables it to interact with other animals. Here, we demonstrate that following a short and simple habituation protocol, mice can be trained to interact with a conspecific while head restrained. The HRMM could not express all the behaviors witnessed in the free arena context. For example, mounting was not possible and chasing was only possible when the female mouse was oriented in the same direction of the male and walked at the same pace. However, we noticed that some of the mice learned to induce close proximity of the female by running and moving the wheel (see Supplementary Movie 2). This behavior, in addition to sniffing and the vocalizations, indicates that even with the head restraint a rich social behavior was achieved.

More than half of the mice that were vocalizing on the first encounter with a female mouse (in a free arena context), could be habituated to vocalize when head restrained. This success rate (60%) is comparable to other studies training mice on a behavioral task while head restrained and makes this protocol feasible for application in experiments recording brain activity during vocalization. Using the criteria based on the analysis of the vocalizations emitted in the first encounter session, we expect that the success rate will be significantly higher, simplifying the process even further. We expect that this will open the door for further studies involving other social interaction paradigms and genetic models known to affect social behavior.

## Author contributions

BW: carried out the experiments. NP: performed early version proof of concept experiments. SH and BW: performed the data analysis. BW and ML: wrote the manuscript.

### Conflict of interest statement

The authors declare that the research was conducted in the absence of any commercial or financial relationships that could be construed as a potential conflict of interest.
