# Supplementary material for: Social Ultrasonic Vocalization in Awake Head-Restrained Mouse
Source: Front Behav Neurosci. 2016 Dec 19;10:236. doi: 10.3389/fnbeh.2016.00236 (PMC5165246; doi:10.3389/fnbeh.2016.00236)
Supplement: Supplementary file 1 [file Table1.DOCX]

**Supplementary material:**

| Parameter | S1 | S4 | P value | T value |
| --- | --- | --- | --- | --- |
| Avg sequence length | 8.4 ± 9.2 syl | 8.7 ± 10.7 syl | 0.5706 | 0.5914 |
| Inter-syllable interval | 822.4 ms ± 12.7013 | 843.3 ms ± 10.8521 | 0.8891 | 0.1394 |
| Inter-syllable interval <300 ms | 94.0ms ± 10.4ms | 89.2 ± 16ms | 0.3159 | 2.6790 |
| Simple syllable length | 16.2ms ± 5.7 ms | 15.7ms ± 7.7ms | 0.9687 | 0.0399 |
| Up syllable length | 45.5ms ± 13.1ms | 31.7ms ± 20.6ms | 0.5794 | 0.5858 |
| Down syllable length | 32.1ms ± 9.2 ms | 30.7ms ± 19.4ms | 0.9935 | 0.0083 |
| Multi syllable length | 63.5ms ± 18.7ms | 58.6ms ± 38.9ms | 0.9877 | 0.0158 |

**Supplementary table 1**: Student's non-paired t-test was used to compare properties of USVs sequences and individual syllable between NR (S1) and HR (S4) sessions across 15 mice (excluding two mute mice of session 1). Note: Values are mean ± standard deviation. No significant change in any of these parameters was detected.

**Supplementary Movie 1**: One minute of recording of male and female mice during session 1 (cage). USVs were down sampled 10 folds to make them audible.

**Supplementary Movie 2**: One minute of recording of male and female mice during session 4 (HRMM). USVs were down sampled 10 folds to make them audible.
